# Repeated Leftover Serosurvey of SARS-CoV-2 IgG Antibodies in Greece, May to August 2020

**DOI:** 10.3390/vaccines9050504

**Published:** 2021-05-13

**Authors:** Zacharoula Bogogiannidou, Matthaios Speletas, Alexandros Vontas, Dimitrios J. Nikoulis, Katerina Dadouli, Maria A. Kyritsi, Varvara A. Mouchtouri, Paraskevi Mina, Lemonia Anagnostopoulos, Michalis Koureas, Vasileios Karavasilis, Olga Nikou, Ourania Pinaka, Pavlos C. Thomaidis, Kornilia Kadoglou, Konstantinos Bedevis, Natalia Spyrou, Alexandros A. Eleftheriou, Vassiliki Papaevangelou, Achilleas Gikas, Alkiviadis Vatopoulos, Evangelia E. Ntzani, Panagiotis Prezerakos, Sotirios Tsiodras, Christos Hadjichristodoulou

**Affiliations:** 1 Laboratory of Hygiene and Epidemiology, Faculty of Medicine, University of Thessaly, 41222 Larissa, Greece; xara.16.01@gmail.com (Z.B.); avontas@uth.gr (A.V.); dnikoulis@uth.gr (D.J.N.); katerina1dad@gmail.com (K.D.); mkiritsi@med.uth.gr (M.A.K.); mouchtourib@med.uth.gr (V.A.M.); pmina@med.uth.gr (P.M.); lanagnost@uth.gr (L.A.); mihaliskoureas@gmail.com (M.K.); karavassilis_v@yahoo.gr (V.K.); onikou@med.uth.gr (O.N.); rpinaka@gmail.com (O.P.); 2 Department of Immunology and Histocompatibility, Faculty of Medicine, University of Thessaly, 41500 Larissa, Greece; maspel@med.uth.gr; 3 Microbiological Laboratory “Thomaidis”, 20131 Korinthos, Greece; thomaidispavlos@gmail.com; 4 Biochemistry Laboratory, General Hospital of Kalamata, 24150 Kalamata, Greece; kadkoveroia@gmail.com; 5 Microbiological Laboratory “Bedevis Konstantinos”, 22100 Tripoli, Greece; kkbed@me.com; 6 Microbiological Laboratory “Bioiatriki”, 11524 Athens, Greece; nataspyrou@gmail.com; 7 Department of Hygiene and Epidemiology, University of Ioannina Faculty of Medicine, 45110 Ioannina, Greece; alexandroseleftheriou4@gmail.com (A.A.E.); entzani@uoi.gr (E.E.N.); 8 Third Department of Paediatrics, School of Medicine, Attikon University Hospital, National and Kapodistrian University of Athens, 12462 Athens, Greece; vpapaev@gmail.com; 9 Internal Medicine Department, Infectious Diseases Unit, University Hospital of Heraklion, School of Medicine, University of Crete, 71500 Heraklion, Greece; gikas.achilles@uoc.gr; 10 Department of Public Health Policy, School of Public Health, University of West Attica, 11521 Athens, Greece; avatopoulos@uniwa.gr; 11 Center for Evidence Synthesis in Health, Department of Health Services, Policy and Practice, School of Public Health, Brown University, Providence, RI 02903, USA; 12 Institute of Biosciences, University Research Center of loannina, 45110 Ioannina, Greece; 13 Department of Nursing, University of Peloponnese, 22100 Tripoli, Greece; prezerpot@gmail.com; 14 Fourth Department of Internal Medicine, School of Medicine, Attikon University Hospital, National and Kapodistrian University of Athens, 12462 Athens, Greece; tsiodras@med.uoa.gr

**Keywords:** SARS-CoV-2, COVID-19, antibodies, IgG, seroepidemiology, long-term immune response

## Abstract

A serosurvey of IgG antibodies against SARS-CoV-2 was conducted in Greece between May and August 2020. It was designed as a cross-sectional survey and was repeated at monthly intervals. The leftover sampling methodology was used and a geographically stratified sampling plan was applied. Of 20,110 serum samples collected, 89 (0.44%) were found to be positive for anti-SARS-CoV-2 antibodies, with higher seroprevalence (0.35%) observed in May 2020. The highest seroprevalence was primarily observed in the “30–49” year age group. Females presented higher seroprevalence compared to males in May 2020 (females: 0.58% VS males: 0.10%). This difference reversed during the study period and males presented a higher proportion in August 2020 (females: 0.12% VS males: 0.58%). Differences in the rate of seropositivity between urban areas and the rest of the country were also observed during the study period. The four-month infection fatality rate (IFR) was estimated to be 0.47%, while the respective case fatality rate (CFR) was at 1.89%. Our findings confirm low seroprevalence of COVID-19 in Greece during the study period. The young adults are presented as the most affected age group. The loss of the cumulative effect of seropositivity in a proportion of previous SARS-CoV-2 infections was indicated.

## 1. Introduction

Severe acute respiratory syndrome coronavirus 2 (SARS-CoV-2) is a betacoronavirus and the infectious agent that causes coronavirus disease (COVID-19), a respiratory infection with systemic involvement. The first documented COVID-19 case occurred in Wuhan, China [1] at the end of 2019. Initially, cases were only recorded in Hubei province; however, the COVID-19 outbreak rapidly evolved and was soon characterized as a pandemic by the World Health Organization (WHO) [2].

The first COVID-19 case in Greece was confirmed on 26 February 2020, marking the start of the first pandemic wave in the country, which began in March and lasted until May 2020. During the summer months, some touristic areas within the country recorded a small increase in cases. By 31 August 2020, Greece had reported 8986 laboratory confirmed cases of SARS-CoV-2 infection in the general population and 266 related deaths [3]. The recorded cumulative incidence of COVID-19 in Greece until 31 August was estimated at 87.2 cases per 100,000 population, and the mortality at 2.6 deaths per 100,000 population. The second pandemic wave in Greece began in October 2020. 

In an effort to manage the pandemic, countries have applied different strategic interventions with varying levels of success [4]. Extensive diagnostic testing and subsequent interventions are considered essential for controlling and interrupting SARS-CoV-2 transmission. A gradual deterioration of the epidemiological situation in Greece resulted in the implementation of a strict lockdown on 23 March 2020, requiring all residents to limit non-essential movement with minimal exceptions. Beginning on 4 May 2020, public health measures were gradually lifted, with retail/trade businesses the first to reopen, followed by the reopening of schools. As of 1 July 2020, Greece opened its points of entry to tourists. 

Measuring host immune response to SARS-CoV-2 infection is an indirect method for detection of COVID-19 beyond the first 2 weeks of illness onset [5]. Considering both the insufficient number of molecular tests conducted and that the majority of individuals infected with SARS-CoV-2 display mild symptoms or remain completely asymptomatic, serological diagnosis is becoming an important tool to understand the extent of COVID-19 in the community. Furthermore, serological diagnosis allows for the identification of individuals who are immune and potentially “protected” from becoming infected. The duration of antibody response, particularly in asymptomatic or mild infections is not yet known. 

Many scientific articles support the idea of antibodies waning and falling below the threshold of seropositivity approximately two to three months after COVID-19 diagnosis [6,7]. According to the Centers for Disease Control and Prevention (CDC), approximately 2 months after an antibody test, 28% of seropositive individuals seroreverted to below the threshold of positivity. Thus, point seroprevalence studies could face challenges related to interpretation of results. Conducting repeated serosurveys on a monthly basis using the same sampling methodology could be considered a supplementary surveillance tool. 

This seroepidemiological study began in March 2020 and has been repeated at monthly intervals. Results from the first two months have been published elsewhere [8], while here we report on the results from May to August 2020.

The aims of the present seroepidemiological study are to provide an assessment of the extent of COVID-19 spread in the community through estimation of prevalence of SARS-CoV-2 IgG antibodies in the Greek population by sex, age group and geographical area; to identify regional, sex and age differences throughout the entire course of the pandemic; and to assess the infection fatality rate (IFR) and compare it to the case fatality rate (CFR). Finally, this serosurvey intends to provide evidence regarding potential under-diagnosis of COVID-19 in Greece.

## 2. Materials and Methods

### 2.1. Study Design and Participants

The study was designed as a cross-sectional survey and repeated at monthly intervals. We used the leftover sampling methodology in order to collect serum samples (residual sera from the general population) [9]. A geographically stratified sampling plan based on regional units (NUTS level 3) was applied in order to produce a representative sample, taking into consideration age group (0–29, 30–49, 50–69, and ≥70 years) and sex. The required sample size was determined to be 380 serum samples from each of the 13 NUTS level 2 regions and the sample size for each regional unit (NUTS level 3) from the corresponding region was calculated according to population distribution. However, the actual number of collected samples differed from the pre-determined number of samples above.

The leftover serum samples were collected from a nationwide laboratory network, including both private microbiological laboratories as well as microbiological and biochemical laboratories of public hospitals. A total of 36 laboratories participated. The samples were derived from individuals who visited the laboratories for routine screening and reasons unrelated to COVID-19.

The majority of private laboratories, particularly in large urban areas, were closed due to the summer holidays in August 2020, resulting in more challenging sample collection.

### 2.2. Laboratory Analysis

The presence of anti-SARS-CoV-2 IgG antibodies was determined using the ABBOTT SARS-CoV-2 IgG assay, a chemiluminescent microparticle immunoassay (CMIA), with the ARCHITECT i2000SR analyzer (Abbott, Illinois, United States). Anti-spike IgG antibodies are used as a marker of prior SARS-CoV-2 infection. As already stated, the method was validated in our laboratory. We used 305 pre-COVID-19 samples (obtained in 2017) as negative controls and 94 samples from patients with positive SARS-CoV-2 PCR and different symptom durations. The kit displayed 84.0% sensitivity (95% confidence interval (CI): 76.6–91.5) and 99.7% specificity (95% CI: 98.2–100). Given that there were not vaccines during the study period (May to August 2020), all positive samples for IgG anti-SARS-CoV-2 were provoked by natural infection.

### 2.3. Statistical Analysis

The statistical analysis applied is identical to the analysis applied for samples between March and April 2020 [8].

### 2.4. Weighted Prevalence 

Initially, we determined an unweighted relative frequency of all patient characteristics (age, sex and area of residence): this is the crude seroprevalence (S1). The weighted proportions of positive tests in the countrywide sample were based on the sex and age distribution within each regional unit (NUTS level 3) and the population of each regional unit, according to the most recent census conducted in 2011 (S2) [10]. We also adjusted the weighted proportion (S2) of positive tests to account for the accuracy (sensitivity and specificity) of the laboratory test (S3) [11,12]. Since reported COVID-19 cases were by definition outside the sampling framework, the seroprevalence was corrected, taking into consideration the number of reported cases per month in accordance with the National Public Health Organization (NPHO) (S4). Therefore, we added the cases reported in March, April and May to the estimated S3 seroprevalence in order to calculate the S4 for May, while to calculate the S4 for June we added the reported cases from March to June, and so forth. We calculated the S1, S2, S3 and S4 seroprevalence of IgG antibodies by month and also calculated the CFR and IFR by month. CFR is the ratio of the number of deaths attributed to COVID-19 and reported to the NPHO, divided by the number of cases reported to the NPHO; IFR is the ratio of deaths divided by the number of estimated individuals infected with SARS-CoV-2. The estimation of infected individuals was the product of the seroprevalence and population of regional units where confirmed cases were identified according to NPHO [13]. The 95% CI for weighted data were estimated using normal approximation of binomial distribution and effective sample size, rather than the collected sample size (further explained below). It should be noted that clusters of cases from refugee camps and from a cruise ferry - which were not considered community cases (302 cases in total) - were excluded from analysis for CFR and IFR. The 95% CI for CFR was calculated using normal approximation of binomial distribution. The 95% CI for IFR was calculated using the corresponding 95% CI of the S1, S2, S3 and S4 seroprevalence. Comparison of two proportions was carried out with the ‘N-1’ chi-squared test [13]. In order to calculate how many SARS-CoV-2 infections correspond to one reported case, we estimated the average S3 seroprevalence and corrected it by the estimated 28% seroconversion according to CDC. For all analyses, a 5% significance level was set. 

### 2.5. Effective Sample Size

Since the number of collected samples from each regional unit was not proportional to the regional unit’s population, we calculated an effective sample size based on each regional unit’s population proportion, according to 2011 census data. This was done using target weighting. The target sample size for a regional unit i is t_i_, and the actual sample size for the regional unit i is a_i_. The weighting factor for the regional unit i is calculated with the following formula:

(1) $$f_{i}=\frac{t_{i}}{a_{i}}\;.$$

The weighted sample size (w_i_) for the regional unit i is calculated as follows:

w_i_ = t_i_ × f_i_. (2)

For k regional units and a countrywide target sample size of n_t_, the country-wide effective sample size (n_e_) is calculated with the following formula:

(3) $$n_{e}=\frac{n_{t}{}^{2}}{\sum_{i=1}^{k}w_{i}}\;.$$

This can also be written as:

(4) $$n_{e}=\frac{{\left(\sum_{i=1}^{k}t_{i}\right)}^{2}}{\sum_{i=1}^{k}\frac{t_{i}{}^{2}}{a_{i}}}\;.$$

### 2.6. Ethical Statement

The samples were anonymized leftover serum samples. Each sample had a unique code and the required data—sex, age, residence and date of blood sampling—were recorded. Health staff from the participating laboratories requested written consent statements from the involved individuals. The research protocol was approved by the ethical committee of the Faculty of Medicine, University of Thessaly, Greece (No. 2116).

## 3. Results

A total of 20,110 samples were collected for the four month period between May and August 2020, of which 11,481 (57%) were obtained from females. Regarding the age distribution of collected samples, 4375 (21.8%) belonged to the “0–29” age group, 5957 (29.6%) to the “30–49” age group, 5328 (26.5%) to the “50–69” age group and 4547 (22.6%) to individuals “>70” years of age. 

**Figure 1:**
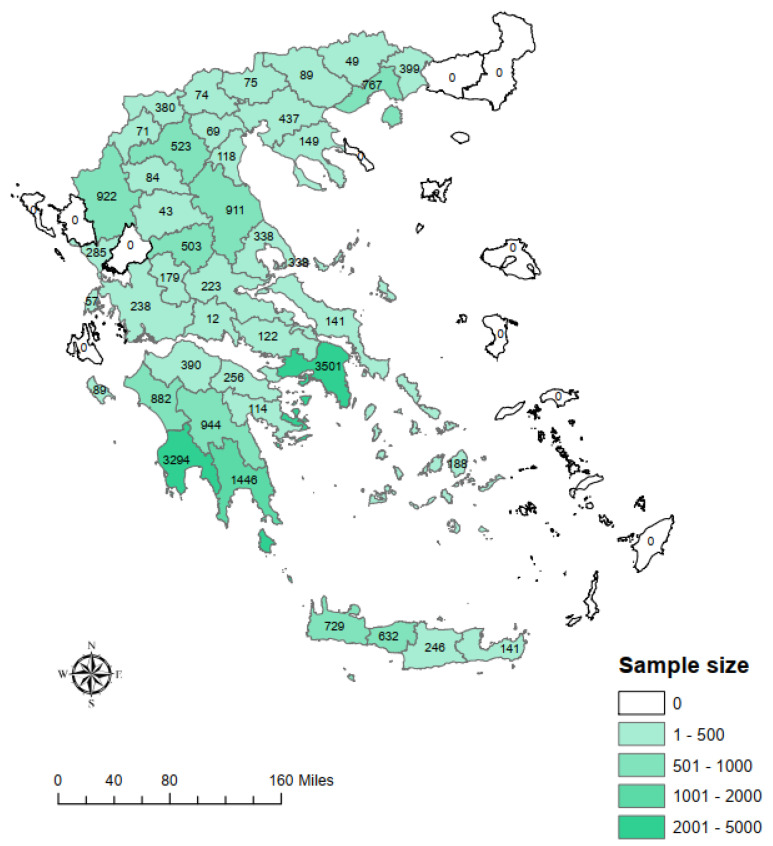
Geographical distribution of leftover samples collected for COVID-19 serosurvey. Some RUs are not covered due to the absence of participating microbiological laboratories.

Figure 1 displays the geographic distribution of collected leftover samples. A total of 6,054 samples were collected from the Peloponnese region, followed by 3501 from Attica, 1795 from Thessaly, 1748 from Crete, 1510 from Western Greece, 1215 from Eastern Macedonia and Thrace, 1207 from Epirus, 1058 from Western Macedonia, 1011 from Central Macedonia, 677 from Central Greece, 188 from South Aegean and 146 from the Ionian Islands. For each sample, age, sex, residence and date of blood sampling were recorded.

Of the 20,110 collected serum samples, 89 (0.44%) were found positive for anti-SARS-CoV-2 IgG antibodies. According to the monthly distribution of samples, S1 seroprevalence for anti-SARS-CoV-2 IgG antibodies was as follows: 0.44% in May, 0.37% in June, 0.49% in July and 0.52% in August (Table 1, Table 2, Table 3 and Table 4). The adjusted results for age, sex, population (S2) and additionally, for accuracy of the laboratory test (S3) are presented in Table 1, Table 2, Table 3 and Table 4. After the addition of NPHO data, S3 seroprevalence was modified and S4 was calculated as 0.35% in May, 0.19% in June, 0.25% in July, and 0.35% in August (Table 1, Table 2, Table 3 and Table 4).

**Figure 2:**
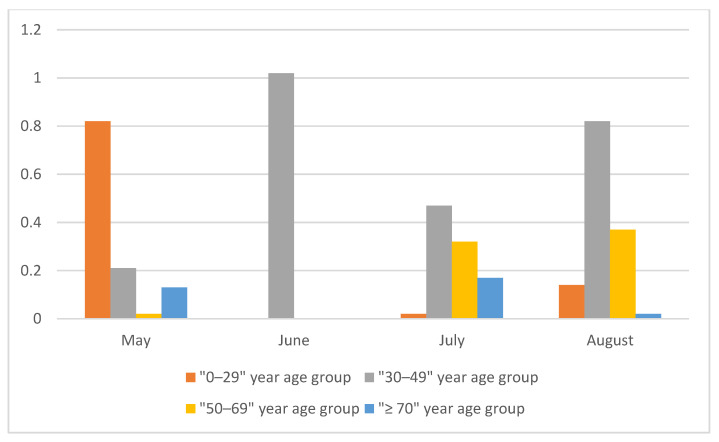
Seroprevalence by age group between May to August 2020.

Throughout the study period, the two younger age groups presented the highest seroprevalence. Specifically, in May 2020 the highest seroprevalence was observed in the “0–29” year age group with S4 = 0.82%, while in June, July and August 2020 the highest seroprevalence was estimated in the “30–49” year age group with S4 = 1.02%, 0.47%, and 0.82%, respectively (Table 1, Table 2, Table 3 and Table 4, Figure 2).

During the first two months of the study period, higher seroprevalence was estimated in large urban areas (Attica region and the regional unit of Thessaloniki) as compared to the rest of the country. In large urban areas, the S4 was calculated as 0.47% and 0.46% in May and June respectively, while in the rest of the country the S4 was calculated as 0.16% and 0% for the corresponding months (May: difference = 0.31%, *p* = 0.039; June: difference = 0.46%, *p* < 0.0001) (Table 1 and Table 2). However, in July 2020 the S4 was calculated as 0.29% in large urban areas and 0.21% for the rest of the country, with no statistically significant difference estimated (*p* = 0.570). Due to an insufficient number of samples collected from large urban areas in month of August, due to closures of many microbiological laboratories during summer holidays, seroprevalence was not calculated separately based on areas by population density. 

**Figure 3:**
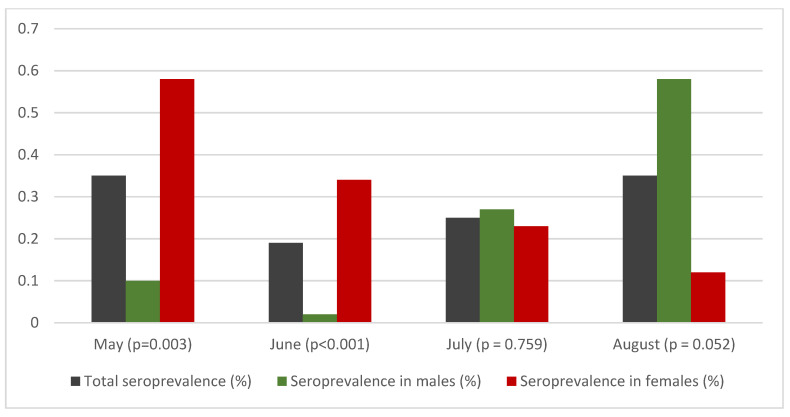
Total seroprevalence, and seroprevalence per sex from May to August 2020 (*p* indicates the difference between sexes).

The S4 was higher among females as compared to males in May and June (May: females: S4 = 0.58% VS males: S4 = 0.10%, June: females: S4 = 0.34%, VS males: S4 = 0.02%) (Table 1 and Table 2); however, this difference was reversed during July and August (July: females: S4 = 0.23% VS males: S4 = 0.27%, August: females: S4 = 0.12% VS males: S4 = 0.58%) (Figure 3). The difference between sexes observed in August (0.46%) is of borderline statistical significance (*p* = 0.052). Additionally, seroprevalence in each age group was calculated separately for each sex (Table S1).

Summarizing the above results, for the months of May and June, statistically significant difference was observed between the two sexes (May *p* = 0.003 and June *p* < 0.001) and between large urban areas and rest of the country (May = 0.039 and June *p* < 0.001) with females and large urban areas presented higher seroprevalence. These differences were not observed in July. In August, a borderline no statistically significant difference was calculated (*p* = 0.052) between males and females, with S4 = 0.58% in males and 0.12% in females.

Table 1, Table 2, Table 3 and Table 4 present the monthly CFR and IFR. A gradual decline in CFR was observed, while IFR remained below 0.2% each month during the study period, presenting a slight difference from month to month. Between May and August 2020, 126 deaths were reported according to data from the NPHO. Consequently, the CFR for this 4-month period was calculated as 1.89% and the corresponding IFR as 0.47% (Table 5). 

A total of 5094 new cases were reported between May and August 2020 in the general population, excluding the Attica region. By using the average S3 seroprevalence for this time period and increasing it by 28% (the percentage of seropositives that seroconvert), according to the CDC it was estimated that each case reported by the NPHO corresponded to 4.9 SARS-CoV-2 infections in the general Greek population (95% CI: 2.1–7.8).

## 4. Discussion

**Figure 4:**
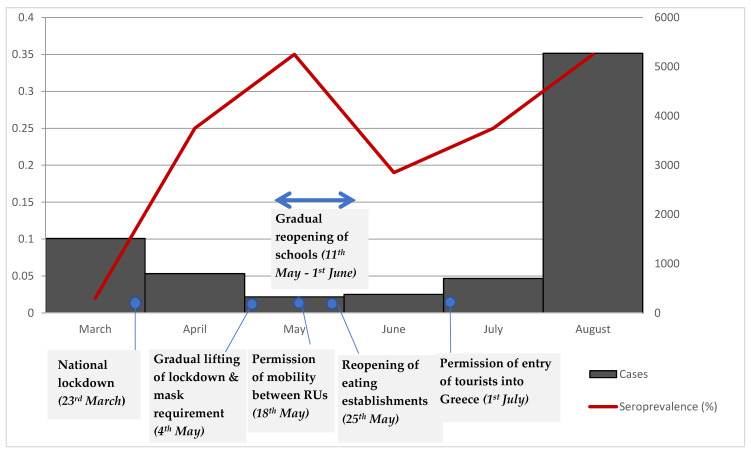
The seroprevalence, monthly cases and main public health measures in Greece during the period from March to August 2020.

Our results demonstrate low seroprevalence of anti-SARS-CoV-2 IgG antibodies in Greece during the first wave of the pandemic. This finding supports hypotheses regarding the effectiveness of the timely implementation of public health measures. In March and April 2020, the serosurvey results were calculated as S4 = 0.02% and 0.25%, respectively [8]. In Figure 4, we summarize our previous and current serosurvey results, in addition to presenting the monthly cases according to NPHO data. A peak in seroprevalence was observed in May 2020; this peak can be explained as representing the proportion of individuals infected with SARS-CoV-2 during the first pandemic wave between March to May 2020. Consequently, an increase in seroprevalence would be expected in May, as compared to March and April 2020. Figure 4 becomes more remarkable, taking into consideration the different public health measures implemented during this period. From 23 March 2020 a national lockdown had been imposed, which was gradually lifted beginning 4 May 2020, with a requirement for masks to be worn in areas of intense crowding (such as on means of public transport, at supermarkets, hospitals etc.). Furthermore, on 18 May mobility between regional units within the country was permitted. Between mid-May to June, schools gradually reopened with senior classes the first to commence. In late May, eating establishments were reopened and starting from 1 July 2020, the entry of tourists into Greece from all countries was permitted.

A cumulative effect of seropositivity would be conventional, increasing even with lower positivity rates over the following months. However, the seroprevalence in June is lower than the seroprevalence in May. This finding has been documented by corresponding seroepidemological studies reflecting antibody waning [6,14].

Our positive results reflect mainly mild or asymptomatic cases, since confirmed COVID-19 cases that occurred during the same month were excluded from the sampling framework. It is widely supported that both anti-SARS-CoV-2 antibody titers and their duration of detection are correlated with the severity of clinical presentation [15]. Mild COVID-19 cases develop antibodies in a lower titer and for a shorter time period. The half-life of anti-SARS-CoV-2 antibodies has been calculated as 106 days [7] and, subsequently, several of them end in “serosilent” infections. 

As mentioned above, the CDC suggests that, approximately 2 months after the first detection of antibodies, nearly one of four seropositive individuals seroconverted below the threshold of positivity [6]. This documented waning of antibodies following infection decreases the sensitivity of antibody detection throughout the months. Considering this, the observed decrease of seroprevalence in June 2020 can be justified by both the reduced spread of SARS-CoV-2 in the community and the simultaneous seroconversion of seropositive individuals infected in March and April 2020.

Τhis decrease in seroprevalence raises concerns related to long-term immune response. A few reports exist that describe cases of reinfection; however, this occurrence is extremely rare [16]. In the following months between July and August 2020, a small increase in seroprevalence was observed. This finding may be explained by the unrestricted movement of citizens during this period and increased travel due to summer holidays.

During the summer months, it is well-recognized and customary for the general Greek population to travel from large urban areas to areas throughout the rest of the country. This “pattern of mobility” may offer an explanation for our second remarkable finding, regarding the diminished differences in seroprevalence between these two areas. In May and June 2020, the calculated seroprevalence was higher in large urban areas compared to the rest of the country, while almost the same seroprevalence was estimated in July 2020 for both areas. This finding reflects the spread of SARS-CoV-2 in non-urban areas due to population movement for summer holidays, after the remaining movement restrictions were lifted for the general population on 25 May 2020 Individuals from large urban areas where higher SARS-CoV-2 spread had been observed travelled to their holiday destinations throughout the rest of country, where the susceptible local populations were at risk of becoming infected. This “population movement” resulted in a lessening of seroprevalence differences between the two areas. 

Unfortunately, a sufficient number of samples from large urban areas for August 2020 were unavailable. However, the existence of a difference in seroprevalence between large urban areas and the rest of the country will be further examined in the following months, as the collection of samples continued from September 2020 onward according to the established methodology. 

In contrast to our previous results presenting a simultaneous increase in seropositivity with increasing age, seroprevalence between May and August 2020 was primarily higher in the “30–49” year age group [8]. This finding could be attributed to the fact that Greece’s workforce is largely comprised of individuals in this age group and, furthermore, increased mobility is associated with this subset of the population due to their more active participation in recreational activities. Considering the above factors and that younger age groups that become infected with COVID-19 typically experience mild disease, may offer an explanation for the high seroprevalence observed in this age group [17]. 

Continuous communications campaigns provided by public health authorities regarding the protection of elderly populations likely influenced the behavior of elderly individuals after restrictive public health measures were lifted. This protective behavior could be considered as a reason for the low seroprevalence observed in the “>70” year age group. The results of the next months will enrich the discussion of this issue.

Throughout the months, an increase in seroprevalence in males compared to females can be observed. This finding is consistent with data from the NPHO (55.6% of cases refer to males). Interpretation of this result must also consider that males comprise a large majority of the workforce in Greece and tend to participate more actively in recreational activities. 

Both findings related to age, group and sex are consistent with the results of a published systematic review and meta-analysis, which included preprints or peer-reviewed articles up to 14 August 2020. Higher seroprevalence is calculated in males compared to females. Moreover, in this review the “20–49” year age group shows the highest prevalence, followed by the “50–65” year age group [18].

A total of 2310 COVID-19 cases were reported by the NPHO up to 30 April 2020, and it was calculated that one reported COVID-19 case corresponded to ten SARS-CoV-2 infections in the Greek general population [8]. For the following months, the corresponding factor decreased to 4.9. This reduction is justified by an increase in the number of tests conducted and therefore, increased detection of COVID-19 cases. A similar ratio of approximately 4 was identified when comparing the estimated CFR with the IFR for this four-month period. It should be noted that the delay of death could not be accounted for in the current estimation.

The leftover sampling methodology could be considered a limitation of the study, as non-random convenient sampling may affect the representativeness of samples collected. A limitation of the leftover sampling methodology is the difficulty related to collection of clinical information. Using this methodology, it was therefore not possible to correlate clinical status with positive samples. Sample collection was also challenging due to summer closures of many microbiological laboratories, primarily in August 2020. Not all areas were covered by the sampling network. 

This methodology has several advantages including ease of sample collection. Furthermore, this methodology allows for repeated monthly sample collection, enabling follow up of the course of the pandemic and immunity levels of the general population on a rolling basis.

## 5. Conclusions

Our study presents low seroprevalence in Greece during the period from May to August 2020, a finding which renders the Greek population extremely vulnerable to SARS-CoV-2 infection. The “30–49” year age group appears to have been most affected during this period, since this subset of the population is characterized by the highest mobility levels and most actively participates in recreational activities. The differences in seroprevelance between large urban areas and other areas throughout the country lessened, and towards the end of the study period, a higher seroprevalence in males was observed. As was expected, a lower IFR was calculated compared to CFR. The waning of anti-SARS-CoV-2 IgG antibodies is supported by our serosurvey results. 

## Figures and Tables

**Table 1:** Anti-SARS-CoV-2 IgG antibody seroprevalence, Greece, May 2020 (*n* = 5718).

May		Positive/Sample Size	S1: Crude Prevalence		S2: Age, Sex and Population-Adjusted Prevalence		S3: S2 + Adjustment for Sensitivity and Specificity		S4: S3 + NPHO Data ^1^	
		n/N	Prevalence (%)	95% CI ^2^	Prevalence (%)	95% CI	Prevalence (%)	95% CI	Prevalence (%)	95% CI
Total		25/5718	0.44	0.27–0.61	0.59	0.28–0.89	0.35	0–0.71	0.35	0–0.71
Age group (years)	0–29	7/1181	0.59	0.15–1.03	0.98	0–2.05	0.82	0–2.09	0.82	0–2.09
	30–49	9/1657	0.54	0.19–0.90	0.47	0–0.97	0.21	0–0.80	0.21	0–0.80
	50–69	5/1608	0.31	0.04–0.58	0.31	0–0.72	0.02	0–0.50	0.02	0–0.50
	≥70	4/1272	0.31	0.01–0.62	0.41	0–1.03	0.13	0–0.87	0.13	0–0.87
Sex	Male	8/2428	0.33	0.10–0.56	0.39	0–0.81	0.10	0–0.61	0.10	0–0.87
	Female	17/3290	0.52	0.27–0.76	0.78	0.33–1.23	0.58	0.04–1.12	0.58	0.04–1.12
‘Ν-1’ chi-squared test Difference between sex			Difference = 0.19% *p* = 0.283		Difference = 0.39% *p* = 0.062		Difference = 0.48% *p* = 0.003		Difference = 0.48% *p* = 0.003	
Large urban areas		7/1372	0.51	0.13–0.89	0.69	0.25–1.14	0.47	0–1.00	0.47	0–1.00
Rest of country		16/4346	0.41	0.22–0.61	0.43	0.04–0.82	0.16	0–0.62	0.16	0–0.62
‘Ν-1’ chi-squared test Difference between large urban areas and rest of country			Difference = 0.10% *p* = 0.623		Difference = 0.26% *p* = 0.230		Difference = 0.31% *p* = 0.039		Difference = 0.31% *p* = 0.039	
**CFR (%)**		**95% CI**	**IFR according to**							
			**S1**		**S2**		**S3**		**S4**	
			**IFR (%)**	**95% CI**	**IFR (%)**	**95% CI**	**IFR (%)**	**95% CI**	**IFR (%)**	**95% CI**
10.74		9.11–12.37	0.08	0.06–0.13	0.06	0.04–0.12	0.10	0.05–NA ^3^	0.10	0.05–NA

^1^ According to NPHO: 326 cases, 35 deaths in May; ^2^ CI: confidence interval; ^3^ NA: not applicable.

**Table 2:** Anti-SARS-CoV-2 IgG antibody seroprevalence, Greece, June 2020 (*n*  =  6135).

June		Positive/Sample Size	S1: Crude Prevalence		S2: Age, Sex and Population-Adjusted Prevalence		S3: S2 + Adjustment for Sensitivity and Specificity		S4: S3 + NPHO Data ^1^	
		n/N	Prevalence (%)	95% CI	Prevalence (%)	95% CI	Prevalence (%)	95% CI	Prevalence (%)	95% CI
Total		23/6135	0.37	0.22–0.53	0.46	0.17–0.74	0.19	0–0.53	0.19	0–0.53
Age group (years)	0–29	4/1366	0.29	0.01–0.58	0.22	0–0.64	0	0–0.40	0	0–0.40
	30–49	10/1885	0.53	0.20–0.86	1.16	0.32–1.99	1.02	0.02–2.02	1.02	0.02–2.02
	50–69	6/1625	0.37	0.07–0.66	0.13	0–0.45	0	0–0.18	0	0–0.18
	≥70	3/1259	0.24	0–0.51	0.04	0–0.28	0	0–0.01	0	0–0.01
Sex	Male	12/2785	0.43	0.19–0.67	0.31	0–0.67	0.02	0–0.44	0.02	0–0.44
	Female	11/3350	0.33	0.13–0.52	0.59	0.13–1.04	0.34	0–0.88	0.34	0–0.88
‘Ν-1’ chi-squared test Difference between sex			Difference = 0.17% *p* = 0.366		Difference = 0.54% *p* < 0.001		Difference = 0.46% *p* < 0.001		Difference = 0.46% *p* < 0.001	
Large urban areas		7/1375	0.51	0.13–0.89	0.68	0.19–1.18	0.46	0–1.05	0.46	0–1.05
Rest of country		16/4760	0.34	0.17–0.50	0.14	0–0.36	0	0–0.07	0	0–0.07
‘Ν-1’ chi-squared test Difference between large urban areas and rest of country			Difference = 0.17% *p* = 0.366		Difference = 0.54% *p* < 0.001		Difference = 0.46% *p* < 0.001		Difference = 0.46% *p* < 0.001	
**CFR (%)**		**95% CI**	**IFR according to**							
			**S1**		**S2**		**S3**		**S4**	
			**IFR (%)**	**95% CI**	**IFR (%)**	**95% CI**	**IFR (%)**	**95% CI**	**IFR (%)**	**95% CI**
4.51		3.42–5.60	0.04	0.03–0.07	0.04	0.02–0.10	0.09	0.03–NA	0.09	0.03–NA

^1^ According to NPHO: 377 cases, 17 deaths in June.

**Table 3:** Anti-SARS-CoV-2 IgG antibody seroprevalence, Greece, July 2020 (*n*  =  5959).

July		Positive/Sample Size	S1: Crude Prevalence		S2: Age, Sex and Population-Adjusted Prevalence		S3: S2 + Adjustment for Sensitivity and Specificity		S4: S3 + NPHO Data ^1^	
		n/N	Prevalence (%)	95% CI	Prevalence (%)	95% CI	Prevalence (%)	95% CI	Prevalence (%)	95% CI
Total		29/5959	0.49	0.31–0.66	0.50	0.24–0.76	0.24	0–0.55	0.25	0.01-056
Age group (years)	0–29	6/1221	0.49	0.10–0.88	0.32	0–0.74	0.02	0–0.52	0.02	0–0.52
	30–49	8/1645	0.49	0.15–0.82	0.69	0.13–1.25	0.46	0–1.13	0.47	0.01–1.14
	50–69	9/1586	0.57	0.20–0.94	0.56	0.03–1.09	0.31	0–0.95	0.32	0.01–0.96
	≥70	6/1507	0.40	0.08–0.72	0.44	0–1.01	0.16	0–0.84	0.17	0.01–0.85
Sex	Male	17/2442	0.70	0.37–1.03	0.52	0.11–0.93	0.26	0–0.75	0.27	0.01–0.76
	Female	12/3517	0.34	0.15–0.53	0.48	0.15–0.82	0.22	0–0.62	0.23	0.01–0.63
‘Ν-1’ chi-squared test Difference between sex			Difference = 0.36% *p* = 0.050		Difference = 0.04% *p* = 0.829		Difference = 0.04% *p* = 0.755		Difference = 0.04% *p* = 0.759	
Large urban areas		10/1593	0.63	0.24–1.02	0.53	0.15–0.91	0.28	0–0.73	0.29	0.01–0.74
Rest of country		19/4366	0.44	0.24–0.63	0.47	0.13–0.81	0.20	0–0.61	0.21	0.01–0.62
‘Ν-1’ chi-squared test Difference between large urban areas and rest of country			Difference = 0.19% *p* = 0.353		Difference = 0.06% *p* = 0.768		Difference = 0.08% *p* = 0.561		Difference = 0.08% *p* = 0.570	
**CFR (%)**		**95% CI**	**IFR according to**							
			**S1**		**S2**		**S3**		**S4**	
			**IFR (%)**	**95% CI**	**IFR (%)**	**95% CI**	**IFR (%)**	**95% CI**	**IFR (%)**	**95% CI**
2.00		1.26–0.38	0.03	0.02–0.04	0.03	0.02–0.06	0.06	0.02–NA	0.05	0.02–1.36

^1^ According to NPHO: 700 cases, 14 deaths in July.

**Table 4:** Anti-SARS-CoV-2 IgG antibody seroprevalence, Greece, August 2020 (*n*  =  2298).

August		Positive/Sample Size	S1: Crude Prevalence		S2: Age, Sex and Population-Adjusted Prevalence		S3: S2 + Adjustment for Sensitivity and Specificity		S4: S3 + NPHO Data ^1^	
		**n/N**	Prevalence (%)	95% CI	Prevalence (%)	95% CI	Prevalence (%)	95% CI	Prevalence (%)	95% CI
Total		12/2298	0.52	0.23–0.82	0.55	0.25–0.86	0.30	0–0.66	0.35	0.05–0.71
Age group (years)	0–29	2/607	0.33	0–0.79	0.37	0–0.86	0.09	0–0.67	0.14	0.05–0.72
	30–49	6/770	0.78	0.16–1.40	0.84	0.20–1.49	0.65	0–1.42	0.82	0.17–1.59
	50–69	3/509	0.59	0–1.25	0.58	0–1.29	0.33	0–1.12	0.37	0.04–1.16
	≥70	1/412	0.24	0–0.72	0.27	0–0.77	0.00	0–0.56	0.02	0–0.58
Sex	Male	7/974	0.72	0.19–1.25	0.74	0.20–1.28	0.53	0–1.17	0.58	0.05–1.22
	Female	5/1324	0.38	0.05–0.71	0.36	0.04–0.68	0.07	0–0.46	0.12	0.05–0.51
‘Ν-1’ chi-squared test Difference between sex			Difference = 0.34% *p* = 0.265		Difference = 0.38% *p* = 0.211		Difference = 0.46% *p* = 0.034		Difference = 0.46% *p* = 0.052	
**CFR (%)**		**95% CI**	**IFR according to**							
			**S1**		**S2**		**S3**		**S4**	
			**IFR (%)**	**95% CI**	**IFR (%)**	**95% CI**	**IFR (%)**	**95% CI**	**IFR (%)**	**95% CI**
1.14		0.58–1.70	0.11	0.07–0.26	0.11	0.07–0.23	0.19	0.09–NA	0.17	0.08–1.16

^1^ According to NPHO: 5273 cases, 60 deaths in August.

**Table 5:** Anti-SARS-CoV-2 IgG antibody seroprevalence, IFR, CFR, Greece, May–August 2020.

May–August	Positive/ Sample Size	S1: Crude Prevalence		S2: Age, Sex and Population-Adjusted Prevalence		S3: S2 + ;Adjustment for Sensitivity and Specificity		S4: S3 + NPHO Data	
	n/N	Prevalence (%)	95% CI	Prevalence (%)	95% CI	Prevalence (%)	95% CI	Prevalence (%)	95% CI
Total	89/20,110	0.44	0.35–0.53	0.46	0.37–0.55	0.19	0.08–0.30	0.26	0.15–0.37
**CFR (%)**	**95% CI**	**IFR according to**							
		**S1**		**S2**		**S3**		**S4**	
		**IFR (%)**	**95% CI**	**IFR (%)**	**95% CI**	**IFR (%)**	**95% CI**	**IFR (%)**	**95% CI**
1.89	1.56–2.21	0.28	0.23–0.35	0.27	0.22–0.33	0.63	0.40–1.51	0.47	0.33–0.84

## Supplementary Materials

The following are available online at https://www.mdpi.com/article/10.3390/vaccines9050504/s1, Table S1: S1 and S1 adjusted for Se & Sp in each age group for each sex separately.

## References

[1] Chan J.F.W., Yuan S., Kok K.H., To K.K.W., Chu H., Yang J., Xing F., Liu J., Yip C.C.Y., Poon R.W.S. (2020). A familial cluster of pneumonia associated with the 2019 novel coronavirus indicating person-to-person transmission: A study of a family cluster. Lancet.

[2] WHO Director-General’s Opening Remarks at the Media Briefing on COVID-19—11 March 2020. https://www.who.int/director-general/speeches/detail/who-director-general-s-opening-remarks-at-the-media-briefing-on-covid-19---11-march-2020.

[3] Greece: Coronavirus Pandemic Country Profile-Our World in Data. https://ourworldindata.org/coronavirus/country/greece#what-is-the-cumulative-number-of-confirmed-deaths.

[4] Policy Responses to the Coronavirus Pandemic-Statistics and Research-Our World in Data. https://ourworldindata.org/policy-responses-covid.

[5] Sethuraman N., Jeremiah S.S., Ryo A. (2020). Interpreting Diagnostic Tests for SARS-CoV-2. JAMA.

[6] Self W.H., Tenforde M.W., Stubblefield W.B., Feldstein L.R., Steingrub J.S., Shapiro N.I., Ginde A.A., Prekker M.E., Brown S.M., Peltan I.D. (2020). Decline in SARS-CoV-2 Antibodies After Mild Infection Among Frontline Health Care Personnel in a Multistate Hospital Network—12 States, April–August 2020. MMWR Morb. Mortal Wkly. Rep..

[7] Buss L.F., Prete C.A., Abrahim C.M., Mendrone A., Salomon T., de Almeida-Neto C., França R.F., Belotti M.C., Carvalho M.P., Costa A.G. (2021). Three-quarters attack rate of SARS-CoV-2 in the Brazilian Amazon during a largely unmitigated epidemic. Science.

[8] Bogogiannidou Z., Vontas A., Dadouli K., Kyritsi M.A., Soteriades S., Nikoulis D.J., Mouchtouri V.A., Koureas M., Kazakos E.I., Spanos E.G. (2020). Repeated leftover serosurvey of SARS-CoV-2 IgG antibodies, Greece, March and April 2020. Eurosurveillance.

[9] Nardone A., Miller E. (2004). Serological surveillance of rubella in Europe: European Sero-Epidemiology Network (ESEN2). Eurosurveilllance.

[10] Naing N.N. (2000). Easy way to learn standardization: Direct and indirect methods. Malays. J. Med. Sci..

[11] Bendavid E., Mulaney B., Sood N., Shah S., Ling E., Bromley-Dulfano R., Lai C., Weissberg Z., Saavedra-Walker R., Tedrow J. (2020). COVID-19 Antibody Seroprevalence in Santa Clara County, California. MedRxiv.

[12] Diggle P.J. (2011). Estimating Prevalence Using an Imperfect Test. Epidemiol. Res. Int..

[13] Campbell I. (2007). Chi-squared and Fisher-Irwin tests of two-by-two tables with small sample recommendations. Stat. Med..

[14] Moshe M., Brown J.C., Flower B., Daunt A., Ward H., Elliott P. (2020). Declining prevalence of antibody positivity to SARS-CoV-2: A community study of 365,000 adults. MedRxiv.

[15] Hansen C.B., Jarlhelt I., Pérez-Alós L., Landsy L.H., Loftager M., Rosbjerg A., Helgstrand C., Bjelke J.R., Egebjerg T., Jardine J.G. (2021). SARS-CoV-2 Antibody Responses Are Correlated to Disease Severity in COVID-19 Convalescent Individuals. J. Immunol..

[16] To K.K.W., Hung I.F.N., Ip J.D., Chu A.W.H., Chan W.M., Tam A.R., Fong C.H.Y., Yuan S., Tsoi H.W., Ng A.C.K. (2020). COVID-19 re-infection by a phylogenetically distinct SARS-coronavirus-2 strain confirmed by whole genome sequencing. Clin. Infect. Dis..

[17] Bajema K.L., Wiegand R.E., Cuffe K., Patel S.V., Iachan R., Lim T., Lee A., Moyse D., Havers F.P., Harding L. (2020). Estimated SARS-CoV-2 Seroprevalence in the US as of September 2020. JAMA Intern. Med..

[18] Rostami A., Sepidarkish M., Leeflang M., Riahi S.M., Shiadeh M.N., Esfandyari S., Mokdad A.H., Hotez P.J., Gasser R.B. (2020). SARS-CoV-2 seroprevalence worldwide: A systematic review and meta-analysis. Clin. Microbiol. Infect..

