# Supplementary material for: Repeated Leftover Serosurvey of SARS-CoV-2 IgG Antibodies in Greece, May to August 2020
Source: Vaccines (Basel). 2021 May 13;9(5):504. doi: 10.3390/vaccines9050504 (PMC8152765; doi:10.3390/vaccines9050504)
Supplement: Supplementary file 1 [file vaccines-09-00504-s001.zip › vaccines-1167780-supplementary.pdf]

Supplementary Material

Table 1: S1 and S1 adjusted for Se & Sp in each age group for each sex separately.

	Month	Age group	Positive	Total	S1	95% CI		S1 adjusted for Se & Sp	95% CI	
Male	May	*0-29*	1	526	0.19%	0.00%	0.56%	0.00%	0.00%	0.31%
		30-49	3	620	0.48%	0.00%	1.03%	0.22%	0.00%	0.87%
		50-69	1	697	0.14%	0.00%	0.42%	0.00%	0.00%	0.15%
		70+	3	585	0.51%	0.00%	1.09%	0.25%	0.00%	0.95%
	June	*0-29*	1	688	0.15%	0.00%	0.43%	0.00%	0.00%	0.16%
		30-49	5	800	0.63 %	0.08%	1.17%	0.3944%	0.00%	1.04%
		50-69	5	720	0.69%	0.09%	1.30%	0.47%	0.00%	1.20%
		70+	1	577	0.17%	0.00%	0.51%	0.00%	0.00%	0.25%
	July	*0-29*	3	513	0.58%	0.00%	1.24%	0.34%	0.00%	1.13%
		30-49	5	538	0.93%	0.12%	1.74%	0.75%	0.00%	1.72%
		50-69	5	708	0.71%	0.09%	1.32%	0.49%	0.00%	1.22%
		70+	4	683	0.59%	0.01%	1.16%	0.34%	0.00%	1.02%
	August	*0-29*	2	267	0.75%	0.00%	1.78%	0.54%	0.00%	1.77%
		30-49	3	280	1.07%	0.00%	2.28%	0.92%	0.00%	2.36%
		50-69	1	240	0.42%	0.00%	1.23%	0.14%	0.00%	1.11%
		70+	1	187	0.53%	0.00%	1.58%	0.28%	0.00%	1.53%
	**Month**	**Age Group**	**Positive**	**Total**	**S1**	**95% CI**		**S1 adjusted for Se & Sp**	**95% CI**	
Female	May	*0-29*	6	655	0.92%	0.19%	1.65%	0.74%	0.00%	1.61%
		30-49	6	1037	0.58%	0.12%	1.04%	0.33%	0.00%	0.88%
		50-69	4	911	0.44%	0.01%	0.87%	0.17%	0.00%	0.68%
		70+	1	687	0.15%	0.00%	0.43%	0.00%	0.00%	0.16%
	June	*0-29*	3	678	0.44%	0.00%	0.94%	0.17%	0.00%	0.77%
		30-49	5	1085	0.46%	0.06%	0.86%	0.19%	0.00%	0.67%
		50-69	1	905	0.11%	0.00%	0.33%	0.00%	0.00%	0.03%
		70+	2	682	0.29%	0.00%	0.70%	0.00%	0.00%	0.48%
	July	*0-29*	3	708	0.42%	0.00%	0.90%	0.15%	0.00%	0.72%
		30-49	3	1107	0.27%	0.00%	0.58%	0.00%	0.00%	0.33%
		50-69	4	878	0.46%	0.01%	0.90%	0.19%	0.00%	0.72%
		70+	2	824	0.245%	0.00%	0.58%	0.00%	0.00%	0.33%
	August	*0-29*	0	340	0.00%	0.00%	0.00%	0.00%	0.00%	0.00%
		30-49	3	490	0.61%	0.00%	1.30%	0.37%	0.00%	1.20%
		50-69	2	269	0.74%	0.00%	1.77%	0.53%	0.00%	1.76%
		70+	0	225	0.00%	0.00%	0.00%	0.00%	0.00%	0.00%
